# Explaining News Trust in Social Media News during the COVID-19 Pandemic—The Role of a Need for Cognition and News Engagement

**DOI:** 10.3390/ijerph182412986

**Published:** 2021-12-09

**Authors:** Ines Kožuh, Peter Čakš

**Affiliations:** Faculty of Electrical Engineering and Computer Science, University of Maribor, 2000 Maribor, Slovenia; peter.caks@um.si

**Keywords:** need for cognition, news engagement, news trust, COVID-19, social media

## Abstract

During the recent COVID-19 pandemic, people have, in many cases, acquired information primarily from social media. Users’ need to stay informed and the intensive circulation of news has led to the spread of misinformation. As they have engaged in news, it has raised the question of trust. This study provides a model on how news trust can be explained through a need for cognition and news engagement. Accordingly, 433 Slovenian social media users participated in our survey. Structural equation modeling revealed that (1) the lower the need for cognition and the more prior knowledge about COVID-19 users have, the more they believe that social media news comprises all facts about the disease; (2) the more users believe that news comprises all essential facts, the more they trust that the news depicts the actual situation about COVID-19 accurately; (3) the more users are interested in engaging with social media news, the more they trust that the actual situation about COVID-19 is depicted accurately. These findings may help authorities to frame messages about COVID-19 effectively. We suggest investing more effort in disseminating new scientific evidence about the disease to contribute to the accurate shaping of knowledge about COVID-19 among social media users.

## 1. Introduction

In health crisis situations, users have perceived social media as one of the credible sources of information [1]. The recent COVID-19 pandemic provoked fear, loneliness, isolation, and the lack of socialization [2], leading people to spend more time on social media to seek information, look for support, and share their feelings [3]. A pleasant experience was evoked [4] and the use of social media increased by 21% globally [5].

Even though recent reports [6] show that only a fifth of European citizens trusts social media, a significant shift in consuming news occurred during the pandemic. People relied primarily on social media to acquire information about COVID-19 [7,8,9], monitored the content related to the pandemic constantly [10], and became dependent on the minute-to-minute constant news flow [11]. This can lead to stress from an overabundance of information, resulting in users having less motivation to verify it [12]. Thus, people are more likely to believe the information in line with their emotions and beliefs rather than factual or objective information [13]. Likewise, they are inclined to trust and care more about the news on social media posted by their friends and relatives than news organizations [14].

In the case of COVID-19, social media users also have difficulties in distinguishing between fact, opinion, propaganda, or bias [13]. COVID-19 is a new disease, and the field has been evolving intensively, so any further information has been considered not as “established scientific fact”, but instead as the “best available evidence” [15]. It has caused a misinformation pandemic called “misinfodemic” [16], where online misinformation led to a spread of the disease. It caused major concern in public health, as the impact of misinformation can reduce the effectiveness of initiatives aimed at citizens’ health and awareness about the disease [17].

The present research is a response to the abovementioned global crisis and an answer to the previous ones [18,19], where it was pointed out that we still know little about the factors that influence whether users actually engage with the news content they encounter [20]. Some research [21,22] recognized a need to stay informed as one of the possible factors, also named as a need for cognition (NFC). It affects users’ patterns of information/news consumption, as well as their efforts to evaluate the quality of the information found [21]. Moreover, previous research found that individuals with low news trust prefer social media as a news source, rather than other mainstream media, and are more likely to engage with online news [23].

Our study aims to examine the relationship between NFC and the level of news trust and compelling mediating effects of news engagement on the relationship between NFC and the resultant news trust during the time of the COVID-19 pandemic.

## 2. Methods

### 2.1. Research Model

**Figure 1:**
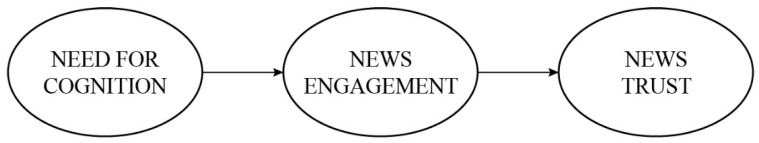
Conceptual framework of the relationship between NFC, news engagement, and news trust.

Very little attention has been devoted to examining how consuming news via different means is associated with news trust in a contemporary media landscape [24]. Accordingly, we proposed a model on how NFC and news engagement may affect users’ trust in news about COVID-19 on social media. The model considers social media news published by all types of users except news organizations. Specifically, we focused on individual-level factors that may affect news trust, as we assumed that news media are not independent of undue business and political influences [24]. Moreover, we assumed that gatekeeping of the content on social media is not necessarily generated by professional journalists, which increases the possibility of misinformation. Concurrently, the focus was on the content regarding COVID-19 that social media users identified with and manifested through creating posts, commenting, sharing, or liking them which is shown in Figure 1. We assumed that users have engaged with such content, i.e., they have read the content or have conducted other related activities, such as thinking about it, liking, sharing, etc.

According to the model, we defined two research questions:

RQ1: How does NFC explain news trust?

RQ2: What is the role of news engagement in explaining the relationship between NFC and news trust?

### 2.2. Research Questions

#### 2.2.1. RQ1: Need for Cognition Explaining News Trust

In what follows, we first define NFC and news trust, and, finally, provide evidence from the literature on how relations between both concepts have already been addressed.

Firstly, NFC is a personality trait that refers to someone’s tendency to enjoy thinking [25]. It also relates to an individual’s need to stay informed, impacting news engagement [22]. Further, it is described as a psychological construct that measures motivation to process a message [26]. Cohen et al. [27] defined it as “a need to structure relevant situations in meaningful, integrated ways”. Such an individual trait is essential for this study, as it helps us understand social media users’ behavior, especially when it comes to consuming social media news. NFC affects the motivation of media usage [28] and gives us an insight into how individuals seek information and how information is needed, used, and shared [21].

Individuals with a high NFC find cognitively demanding tasks enjoyable and are more likely to engage in debates and analyze their solutions. They also tend to focus on relevant arguments in a message and use various information sources to evaluate the quality and credibility of information given in a message [21]. In contrast, those with low NFC have less interest in analytical information processing, preferring quick and superficial evaluations of messages [26]. A lack of critical thinking and verifying information can cause individuals to believe in misinformation that is particularly present and shared on social media. In our study, we considered NFC primarily as a predecessor of news trust, when people use social media to retrieve the news in social media content use.

Secondly, in Western literature, trust has been considered as a foundation of social order and cohesion that often determines a nation’s well-being and its ability to organize and compete [29]. Moving to different types of trust (in government, news, institutions, etc.), there is no unified understanding of how media trust should be defined or measured [30]. However, based on the theory review, the concept of trust in media, in general, can manifest firstly through (1) the believability of news media organizations, (2) the credibility of news media, and (3) the trust in news media as an institution [29]. Secondly, trust can be established through content levels, those delivering the content, and media ownership [31].

As in news engagement, we continue to use content-driven perception at the level of news trust. From the literature, we derived evidence indicating that individuals are more likely to trust and engage with online news when it is shared by someone that they have a higher level of trust in [32]. Thus, we deemed news trust as consisting of four dimensions [33]. Out of these, we considered three dimensions: Trust in the selectivity of topics, trust in the selectivity of facts, and trust in the accuracy of depictions. We excluded the dimension “trust in journalistic assessment”, which is primarily opinion-expressing journalistic content in news organizations’ products that were not the subject of our research. This could turn our focus from the content to emotional or habitual reactions. To conclude, in our study, we were interested in the users’ belief that social media news includes all essential facts, information, and points of view of COVID-19. In addition, we were interested in the users’ perceptions of whether the actual happening regarding COVID-19 was presented accurately in social media news, as well as whether the source of the information was appropriate [33].

Thirdly, as a motivational disposition, NFC has been linked to various fields of media content, including trust in the news [34] or marketing-related media surveys. The literature suggests that high vs. low NFC differs in media attitudes, TV orientation, and media usage [35].

#### 2.2.2. RQ2: Need for Cognition Explaining News Trust through News Engagement

We can conceptualize news engagement on social media elementarily through users’ prior knowledge and interest in a topic [22], in our case, COVID-19. Accordingly, we adopted a content-dependent perception of explaining news engagement on social media, driven mainly by the interestingness of the article and prior knowledge and less by the feelings toward the spreader [22].

Further, news engagement through news content engagement could be defined as a degree of interest and involvement in specific news topics [36] and not necessarily through news medium engagement [37]. While Strömbäck et al. [38] substantiated knowledge as a strong predictor of active news use, Leonhard et al. [39] revealed that some users are less willing to invest actively into staying informed about current news. On the contrary, Chen and Pain [40] identified the concept of social media news engagement regardless of topic-related dimensions, reducing it to two dimensions: content-interaction engagement and exposure engagement. We cannot limit it to users’ habits, i.e., solely as “action-driven” social media news engagement. Instead, it should be considered from the interest in the topic, as users’ content-dependent perceptions of the news posts are the prime driver of news engagement decisions [22].

When considering the relationship between NFC and news engagement, we researched a previous study [20] indicating that the factors influencing users’ engagement with the news content they encounter still lack more detailed research.

### 2.3. Ethics and Procedure

Prior to collecting the data, we obtained the ethical approval of The Institutional Review Board (IRB) of the University of Maribor, Slovenia. The study was also designed in line with the Declaration of Helsinki by the World Medical Association [41] and the Ethical Guidelines released by the Association of Internet Researchers [42]. In January 2021, we invited social media users in Slovenia to participate in an online survey. It was circulated in various public and private social media groups. We were collecting the data between 4 January 2021 and 28 February 2021.

### 2.4. Measuring Instrument

The measuring instrument was an online survey questionnaire comprising two main parts—a demographic part and a part about the variables of the model. In the first part, we asked participants about their age, education, social media use, and COVID-19 self-experience. In the second part, we measured NFC [28], news engagement [22], and news trust [33]. Each was measured with a set of items administered with 5-point Likert-type response categories ranging from 1 = “strongly disagree” to 5 = “strongly agree.” In news engagement, we asked participants to express to what extent they are interested in social media news in symptoms, effects, consequences, and statistical spread of COVID-19. We asked them about their interest in experiences and measures to prevent the spread of the disease. We also measured their prior knowledge in this regard.

In news trust, we measured further the participants’ belief on whether social media news includes all essential and important facts, information, and points of view. They also reported whether they perceived that the actual events surrounding COVID-19 were presented accurately and whether the source of information was appropriate.

## 3. Results

### 3.1. Sample

The target population was adult social media users (aged ≥18 years) residing in Slovenia. The convenience sampling method was selected. In the study, 433 respondents participated. Table 1 shows their demographic characteristics.

### 3.2. Data Analysis

#### 3.2.1. Data Validity and Reliability Analysis

Prior to statistical analyses, we conducted data screening. We proceeded with the confirmatory factor analysis, through which the results confirmed that the construct “news engagement” comprised two variables, the construct “NFC” had one variable, and “news trust” had two variables (see Table 2). Data validity analysis revealed some convergent validity issues; thus, we excluded items with factor loadings lower than 0.5 [43]. Specifically, we dropped one item in the variables “NFC”, “NTaccuracy”, “NEinterest”, and “NEknowledge”. Table 2 shows the remaining items, along with factor loadings and Cronbach’s alpha coefficients, indicating that the internal consistency of the constructs was suitable [44].

We inspected the model fit further, which was achieved successfully (see Table 3). The calculated values were in line with the recommended values, the only marginal value was the normed fit index (NFI), which can still be deemed as acceptable.

A validity and reliability analysis of the model followed. We calculated the composite reliability (CR), average variance extracted (AVE), and the factor correlations matrix (see Table 4). We found no reliability and validity concerns. Both CR and AVE values exceeded the recommended minimum values of CR > 0.7 and AVE > 0.5 [43]. The common method bias was inspected with Harman’s single factor test, where no concerns were found [45]. All statistical analyses were performed with the IBM SPSS Statistics 27.0 and AMOS 27.0 software.

#### 3.2.2. Structural Equation Modeling

In the next step, we checked the linear correlation between items within each construct. No issues were found. Following this, the final structural model was developed. An analysis of the model fit revealed that all values matched the recommended values, and the only marginal values were GFI and NFI (Table 5). Afterward, we tested the validity and reliability of the final structural model. The values for AVE and CR were mostly acceptable (AVE > 0.5 and CR > 0.7), and only a few minor discrepancies were found. Constructs with AVE values below the threshold were NFC (AVE = 0.43), NEknowledge (AVE = 0.39), and NEinterest (AVE = 0.44). Further, there was only one construct with a CR value below the threshold: NFC (CR = 0.64).

### 3.3. The Final Model

**Figure 2:**
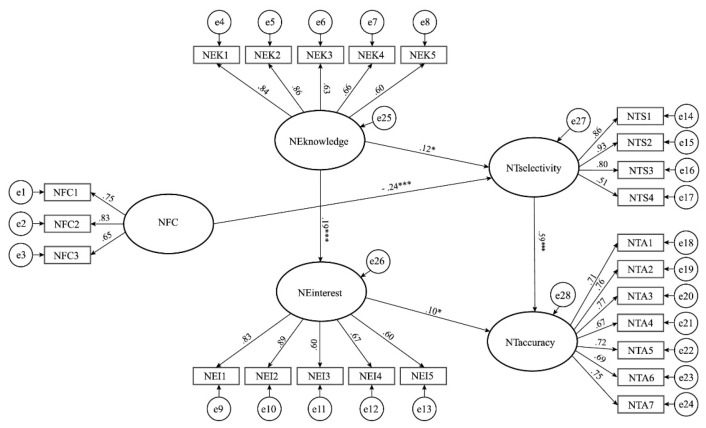
The final model of the relationship between NFC, news engagement, and news trust (Significance level: * *p* < 0.05; *** *p* < 0.001).

Figure 2 shows the final model, where all paths between the variables are statistically significant. The latent variables included in the model explained 64.49% of the variability of the final dependent variable NTaccuracy.

The model suggests a negative and weak but statistically significant effect of NFC on NTselectivity. This indicates that the higher NFC social media users demonstrate, the less they trust that social media news about COVID-19 comprises all essential facts about COVID-19. On the contrary, we found that the variable NTselectivity further affected the final variable NTaccuracy moderately. The effect was positive. This indicates that the more that users trust that social media news comprises all the important information and facts about COVID-19, the more they trust that social media news depicts the situation about COVID-19 accurately. Moreover, we found a positive and weak but statistically significant effect of the variable NEknowledge on the variable NTselectivity and the variable NEinterest. This indicates that the more prior knowledge about COVID-19 users has (symptoms, effects, and consequences of the disease, its statistical spread, experience, and measures to prevent the spread of the disease), the more they trust that social media news comprises all the important information and facts about COVID-19. Likewise, the higher the prior knowledge is, the more interest users have in engaging with social media news about COVID-19, i.e., reading and performing further actions, such as sharing. Finally, we found a positive and weak but statistically significant effect of NEinterest on NTaccuracy, which indicates that the more users are interested in engaging with social media news about COVID-19, the more they trust that social media news depicts the situation about COVID-19 accurately.

## 4. Discussion

This study aimed at examining the mediating effects of news engagement on the relationship between the NFC and news trust. Our first finding revealed that the lower the NFC and the more prior knowledge about the disease COVID-19 social media users have, the more they believe that social media news comprises all essential facts about COVID-19. Consequently, they also trust that this news depicts the situation accurately.

Surprisingly, possessing more prior knowledge about COVID-19 leads to more trust in social media news on this topic. Our study focused on news published by all types of users except news organizations, so there are two possible explanations.

The first possible explanation is that better prior knowledge leads users to be selective, and, consequently, they use credible social media news. In this regard, Kalogeropoulos et al. [24] discovered that using social media as the main source of information is associated with lower levels of trust in news. Similarly, Leding and Antonio [46] revealed that users with high NFC are less inclined to accept misinformation because they engage in more elaborative thinking and may thus detect misinformation, which may be due to possessing more media literacy skills [47]. The second possible explanation is that it may lead them to remain in the like-minded social media bubble they share with other users who publish content they trust. Additionally, their pre-existing attitudes, such as rejection of scientific evidence, may determine their level of belief in misinformation, and belief that they are best informed about the issue [17].

This argument can be supported further by our second finding, in which we found that a lower NFC leads to a higher level of trust in the news regarding the selectivity of facts and information. It seems that users’ trust in social media news is formed without putting much effort into thinking about the content. This is in line with previous research, where higher NFC was found to demonstrate greater skepticism toward information shared on social media, while also reducing beliefs in conspiracy theories [48]. Our finding also complies with Xiao et al. [47], who found that individuals with low NFC and low social media use were least likely to consume new media content critically. As the authors focused on young adults only, our study complements their findings, due to the broader population included in the sample.

Our second finding can be further explained with the media dependency theory. It substantiates that, during the severe social disruption that occurred in the recent health crisis, people usually have had an unusually high need for information, and the mass media have had the potential to satisfy these needs best [2]. However, the emergence of distributed platforms such as social media has disrupted the business of news [24]. Along with changing habits, we have faced the problem of new gatekeepers [49]. These are no longer journalistic professionals, but every individual who contributes to social media through so-called user-generated content. They become opinion leaders who shape users’ news trust significantly [50], which has become even more evident in the pandemic era. We know that users usually engage in the news about COVID-19 published on social media by their network, where they naturally tend to trust other people’s opinions and information credibility [51]. Consequently, they may see online information as more reliable [52], although misleading. On the contrary, using online non-mainstream news sources (digital-born news websites, social media) apart from the combination of traditional news sources (TV, print, and their websites), thus, deeming social media as a main source of information, was found to be associated with lower levels of trust in the news [24].

Another important aspect is that even if we know that higher prior knowledge about COVID-19 leads to a higher level of trust in social media news, we do not know where or how the knowledge acquisition occurred. In the case of COVID-19, the most credible sources of information were found to be the online press and television, followed by institutional websites. On the contrary, Facebook has been recognized as one of the least credible sources [10]. Intriguingly, previous research [9] revealed that the general public and professional public draw information about COVID-19 primarily from social media. As these users may rely heavily on acquiring news in social media, they may develop habits that generally include only seeing small pieces of news, such as short, illustrated teasers linking to original articles. Consequently, they do not read the whole article, which does not lead to processing more complex information [22]. As a result, the acquired knowledge may be a mixture of journalistic actors and algorithmic filters, strategic communicators, social contacts, and users [53].

Given that “the epistemological magnitude of online fake news is detrimental to knowledge acquisition” [54], it may be that the prior knowledge includes false beliefs, which further leads to a higher level of trust even though the news is not trustworthy. These claims are not negligible, as our findings revealed that the more prior knowledge about COVID-19 users have, the more they are interested in news about it. Likewise, the higher the interest, the higher the trust in the accurate presentation of COVID-19 in social media news.

The main limitation of the current study is that the convenience sample was limited to Slovenian social media users. The collection of data was limited, as we were conducting a survey related to COVID-19. Therefore, we were not allowed to advertise the survey, due to current policies that prohibit promoting the content about COVID-19 [55]. In the future, it would be intriguing if the study were repeated on a larger scale. One could balance the sample in distinguishing between users of various types of social media, while distinguished sources of social media news could also be considered.

The next limitation of our study refers to our methodological approach. Even though we used SEM, which can be deemed advantageous, caution must be taken when interpreting our results, as they may have differed if another approach would have been applied. For instance, a causal reasoning framework could be developed, where the assumptions would be translated into counterfactual notation [56,57,58,59].

In summary, we conducted a step forward from the existing empirical evidence about dealing with correlations between media use and trust [34]. The lack of trust in traditional media was found to be related to seeking alternative sources and a higher trust related thereto. Moreover, the rise of news use was found to be present among those who already have a higher level of trust in legacy media and those who are more concerned about the impact of the COVID-19 pandemic [11].

## 5. Conclusions

Our study answers one of the currently most urgent calls of important institutions worldwide, such as the World Health Organization [60] and the European Commission [61], to tackle the spread of misinformation about COVID-19. We provided a model on how news trust can be explained through NFC and news engagement.

Accordingly, our study first revealed whether users’ trust in social media news is determined by users’ personal traits, such as NFC, and their engagement in the news. Our main scientific contribution is the finding that the trigger of users’ interest in engaging in the news about COVID-19 on social media is not their NFC but rather the prior knowledge they already have. Accordingly, it might be that people with high NFC expose themselves superficially to news on social media, but when more complex actions occur, such as through reading or sharing the content, prior knowledge about the topic of news takes the main role instead of NFC.

Our second finding revealed that a user’s decrease in NFC leads to an increase in the belief that news includes all the important information and facts about COVID-19. This finding adds even greater importance in the formation of previous knowledge, as the upgrade of knowledge may be highly dependent on social media algorithms formed upon an individual’s prior activities and interests.

Finally, our findings revealed that, when users’ prior knowledge about COVID-19 extends, their trust that social media news depicts the situation about COVID-19 accurately increases. This could be an important answer to our question about previous knowledge origins, as it points out the self-confidence of social media users when considering sensitive topics, such as that of a public health crisis.

Our study has theoretical and practical contributions. First, in terms of trust research, our attempt was to introduce the concept of news trust. As it is not defined uniformly in the literature, we have drawn a certain frame of the concept and developed it in the context of the current societal situation. As such a health crisis situation may be repeated in the future, our framework may serve as a platform for further adaptations of the concept in new crisis situations. Our next contribution is highlighting the interaction between NFC, news engagement, and news trust. Accordingly, they offer an opportunity to formulate new questions to explore the phenomenon of misinformation spread on social media, as they intersect with socio-political realities.

Moreover, we introduced the concept of NFC, which was already examined in existing research, in relation to patterns of media use, attitudes, and media content. We connected NFC to both news trust and news engagement concurrently for the first time while relying on the previous research [20], where the call for more detailed research of the factors influencing engagement on social media was asserted. Our methodological approach allowed a unique ability to examine a set of dependency relationships and simultaneously analyze the included dependent variables [62]. That is the methodological strength of our study, as we used the structural equation modeling when including NFC, news engagement, and news trust in a model.

In practice, our findings may support policymakers and practitioners in communicating information regarding COVID-19 with the public. As our results stressed the importance of prior knowledge about COVID-19, new scientific evidence about the disease should be disseminated on social media, by media organizations, as well as in various forms and channels offered to reach the required efficiency. We should consider again that the gatekeepers of social media can be all the stakeholders in one’s like-minded social media bubble, which is firstly shaped by continuously changing algorithms, and filled further with original or reposted content by counterparts. Taking into consideration all these factors may help us determine how “accurate” shaping of knowledge in social media users might occur.

## Figures and Tables

**Table 1:** Demographic characteristics of the sample.

Characteristic	Sub-Characteristic	Percentage (%)
Gender	Male	37.6
	Female	62.4
Age	18–24 years	33.9
	25–34 years	25.9
	35–44 years	19.4
	45–54 years	14.3
	55–64 years	6.5
Level of education	Primary level	1.2
	Secondary level	38.8
	Tertiary level	60.1
Social media type	Facebook	100
	Instagram	72.7
	Snapchat	37
	LinkedIn	29.3
	Twitter	24.7
	Other	9.7
Acquiring COVID-19	Yes	18.2
	No	62.1
	Do not know	18.7
	Do not want to answer	0.9

**Table 2:** Reliability of variables and factor loadings for the items.

Construct	Variable	Abbreviation of Variable	Cronbach’s Alpha Coefficient	Item	Factor Loading
Need for cognition	/	NFC	0.790	NFC1	0.74
				NFC2	0.85
				NFC3	0.65
News Engagement	Interest	NEinterest	0.846	NEI1	0.83
				NEI2	0.88
				NEI3	0.61
				NEI4	0.67
				NEI5	0.60
	Prior knowledge	NEknowledge	0.840	NEK1	0.84
				NEK2	0.87
				NEK3	0.63
				NEK4	0.66
				NEK5	0.59
News Trust	Selectivity of facts	NTselectivity	0.846	NTS1	0.86
				NTS2	0.93
				NTS3	0.80
				NTS4	0.51
	Accuracy of depictions and source assessment	NTaccuracy	0.888	NTA1	0.71
				NTA2	0.76
				NTA3	0.78
				NTA4	0.68
				NTA5	0.73
				NTA6	0.69
				NTA7	0.75

**Table 3:** Results of testing the model fit.

Notation.	Recommended Value	Calculated Value
X2		677.598
DF		244
Cmin/df	≤3.0	2.8
RMSEA	≤0.10	0.065
GFI	≥0.90	0.874
NFI	≥0.90	0.872
CFI	≥0.90	0.913

Abbreviations: X2, chi-square value; DF; Cmin/df, chi-square value/degrees of freedom; RMSEA, root-mean-square error of approximation; GFI, goodness-of-fit index; NFI, normed fit index; CFI, comparative fit index.

**Table 4:** Results of validity and reliability analysis of the model.

Notation	CR	AVE	NEknowledge	NEinterest	NTselectivity	NTaccuracy	NFC
NEknowledge	0.846	0.530	**0.728 ^1^**				
NEinterest	0.844	0.527	0.185	**0.726 ^1^**			
NTselectivity	0.863	0.621	0.105	0.181	**0.788 ^1^**		
NTaccuracy	0.888	0.531	0.006	0.203	0.601	**0.729 ^1^**	
NFC	0.793	0.564	0.107	0.084	−0.227	−0.135	**0.751 ^1^**

^1^ The square roots of AVE are the diagonal elements in bold. Abbreviations: CR, composite reliability; AVE, average variance extracted.

**Table 5:** Results of testing the model fit of the final structural model.

Notation	Recommended Value	Calculated Value
X2		696.532
DF		247
Cmin/df	≤3.0	2.82
RMSEA	≤0.10	0.065
GFI	≥0.90	0.872
NFI	≥0.90	0.868
CFI	≥0.90	0.91

Abbreviations: X2, chi-square value; DF, degrees of freedom; Cmin/df, chi-square value/degrees of freedom; RMSEA, root-mean-square error of approximation; GFI, goodness-of-fit index; NFI, Normed fit index; CFI, comparative fit index.

## Data Availability

The raw data supporting the conclusions of this article are freely, available on https://github.com/ikozuh/MDPI-database---Ko-uh-ak-.git (accessed on 5 December 2021).
